# Comparative Analysis of Tunisian Sheep-like Virus, Bungowannah Virus and Border Disease Virus Infection in the Porcine Host

**DOI:** 10.3390/v13081539

**Published:** 2021-08-04

**Authors:** Denise Meyer, Alexander Postel, Anastasia Wiedemann, Gökce Nur Cagatay, Sara Ciulli, Annalisa Guercio, Paul Becher

**Affiliations:** 1 EU and OIE Reference Laboratory for Classical Swine Fever, Institute of Virology, University of Veterinary Medicine Hannover, Foundation, Buenteweg 17, 30559 Hannover, Germany; denise.meyer@tiho-hannover.de (D.M.); alexander.postel@tiho-hannover.de (A.P.); anastasia.wiedemann@tiho-hannover.de (A.W.); goekce.cagatay@tiho-hannover.de (G.N.C.); 2 Department of Veterinary Medical Sciences, University of Bologna, Viale Vespucci, 2, 47042 Cesenatico, Italy; sara.ciulli@unibo.it; 3 Istituto Zooprofilattico Sperimentale della Sicilia “A. Mirri”, Via Gino Marinuzzi, 3, 90129 Palermo, Italy; annalisa.guercio@izssicilia.it

**Keywords:** *Flaviviridae*, pestivirus, Tunisian sheep-like virus, border disease virus, Bungowannah virus, classical swine fever virus, host range, cross-neutralization, antigenic relatedness, phylogenetic analysis

## Abstract

Apart from the established pestivirus species *Pestivirus A* to *Pestivirus K* novel species emerged. Pigs represent not only hosts for porcine pestiviruses, but are also susceptible to bovine viral diarrhea virus, border disease virus (BDV) and other ruminant pestiviruses. The present study focused on the characterization of the ovine Tunisian sheep-like virus (TSV) as well as Bungowannah virus (BuPV) and BDV strain Frijters, which were isolated from pigs. For this purpose, we performed genetic characterization based on complete coding sequences, studies on virus replication in cell culture and in domestic pigs, and cross-neutralization assays using experimentally derived sera. TSV forms a distinct phylogenetic group more closely related to *Pestivirus C* (classical swine fever virus, CSFV) than to *Pestivirus D* (BDV). In contrast to BDV and BuPV, TSV replicates by far more efficiently on ovine than on porcine cells. Nevertheless, pigs were susceptible to TSV. As a consequence of close antigenic relatedness of TSV to CSFV, cross-reactivity was detected in CSFV-specific antibody assays. In conclusion, TSV is genetically closely related to CSFV and can replicate in domestic pigs. Due to close antigenic relatedness, field infections of pigs with TSV and other ruminant pestiviruses can interfere with serological diagnosis of classical swine fever.

## 1. Introduction

Over the last decades several pestiviruses that are genetically distinct from bovine viral diarrhea virus type 1 (BVDV-1, *Pestivirus A*), BVDV-2 (*Pestivirus B*), classical swine fever virus (CSFV, *Pestivirus C*), and border disease virus (BDV, *Pestivirus D*) were discovered in ruminants, pigs, and more recently in non-ungulate hosts. Consequently, the nomenclature and taxonomy of pestiviruses, which belong to the family *Flaviviridae*, were updated [1]. After this last update, novel tentative pestivirus species were detected in pigs, ruminants, bats, rodents, whales and in pangolins [2,3,4,5,6]. 

Pestiviruses of cloven-hoofed animals are widely distributed pathogens and are responsible for economically important diseases affecting domestic and wild animal species worldwide. After infection of ruminant and porcine hosts, the course of the disease can range from subclinical to wasting to fatal. BVDV-1, BVDV-2, and BDV can infect cattle, sheep, goats, other ruminants as well as domestic pigs and wild boar [7,8,9,10,11,12,13,14,15,16]. In contrast, the natural host range of the widely distributed porcine pestiviruses CSFV and atypical porcine pestivirus (APPV) is restricted to domestic pigs and wild boar [17,18]. For other porcine pestiviruses, like Bungowannah pestivirus (BuPV) and Linda virus, only unique reports exist [6,19], and the natural host range of these viruses remains unknown. In contrast to CSFV, postnatal infections of pigs with ruminant pestiviruses frequently do not result in clinical disease and are mainly associated with reproductive disorders. After trans-placental infection of sows, the consequences for health of the offspring depend on the stage of gestation. In particular, the occurrence of persistently infected piglets has a major impact on the epidemiology of the disease since these animals are viremic and antibody negative and excrete virus constantly [8,11].

Due to the antigenic relatedness of CSFV and ruminant pestiviruses, the infection of pigs with ruminant pestiviruses can result in the production of cross-reacting antibodies, which may interfere with the serological diagnosis of classical swine fever (CSF). As an example, the BDV strain Frijters, as well as the BDV strain FNK2012-1, were detected in pigs during the course of CSF sero-surveillance studies [9,10,14]. Especially, pestiviruses from sheep and goat that are even more closely related to CSFV than to other ruminant pestiviruses, e.g., Aydin-like pestivirus (*Pestivirus I*) and the novel ovine pestivirus from Italy (ovIT PeV) (unclassified) can significantly interfere with the serological diagnosis of CSF [20,21,22,23]. Based on this, it is of high importance to characterize novel pestiviruses genetically and antigenically and to evaluate their host range. For the Aydin pestivirus as well as for the recently described ovIT PeV, it was confirmed that piglets could be experimentally infected with these viruses [20,23]. Piglets infected with the ovIT PeV showed only mild clinical signs (e.g., wasting and polyarthritis) and viral replication was confirmed by genome detection in the blood [23]. Infection of piglets with pestivirus strain Aydin did not result in clinical disease and viremia [20]. So far, there are no reports of natural infections of pigs with Aydin-like pestiviruses or the recently described ovIT PeV. 

The Tunisian sheep-like virus (TSV), which was first isolated from batches of a contaminated sheep pox vaccine in Tunisia, represents an additional ovine pestivirus that is genetically closely related to CSFV [24,25]. Subsequently, TSV was also detected in sheep in France as well as in goats and sheep in Italy [26,27]. Analyses of pestivirus evolution resulted in the hypothesis that CSFV emerged from TSV by a host switch from sheep to pig around 225 years ago and that TSV represents not only an ancestor for CSFV but also for the novel ovIT PeV [22,28]. So far, TSV has not been found in pigs and it is unknown whether it has the capacity to cause disease in pigs.

The present study provides a comparative analysis of TSV, BDV and BuPV. For detailed genetic characterization, the complete coding sequences of two TSV isolates from Italy and of BDV strain Frijters, which has been isolated from a pig, were determined and analyzed in the context of known pestivirus species. Moreover, viral replication of TSV, BDV and BuPV was characterized in vitro using different porcine, ovine and bovine cell lines. In addition, it was investigated whether these pestiviruses are able to establish productive infections in pigs under experimental conditions. Finally, the antigenic relatedness of these three pestiviruses was analyzed by cross-neutralization tests (cross NT) and the interference with CSFV-specific serological assays was determined. 

## 2. Materials and Methods

### 2.1. Cells and Viruses

For the analysis of viral in vitro replication, the porcine kidney cell lines PK-15 and SK6, the swine testis cell line STE, the sheep fetal thymus cell line SFT-R and the bovine cell line Madin–Darby bovine kidney (MDBK) were used. For cross NT, PK-15 (infections with CSFV strains, BDV-1 strain Frijters and BuPV), SFT-R (infections with TSV isolate 70282/2007/EN, BDV-3 strain Gifhorn and pestivirus strain Aydin) as well as the porcine embryonic kidney (SPEV) cells (infection with APPV) were applied. 

All cell lines were grown in Eagle’s minimum essential medium (EMEM) without antibiotics supplemented with 10% fetal calf serum (FCS). After viral infection EMEM with antibiotics was used. Before usage, FCS has been tested free for pestivirus genome and antibodies.

The cell lines SFT-R (CCLV-RIE 43), SPEV (CCLV-RIE-0008), STE (CCLV-RIE 255) and PK-15 (CCLV-RIE 5-1) originated from the Collection of Cell Lines in Veterinary Medicine (CCLV, Friedrich-Loeffler-Institute, Island Riems, Greifswald, Germany). The Institute of Virology and Immunoprophylaxis, Mittelhäusern, Switzerland kindly provided the SK6 cell line. The MDBK cell line was obtained from the American Type Culture Collection (Rockville, MD, USA).

All CSFV strains were obtained from the CSF virus collection of the EU and OIE Reference Laboratory for CSF (Institute of Virology, University of Veterinary Medicine, Foundation, Hannover, Germany). The caprine TSV isolate 92019/2007/AG and the ovine TSV isolate 70282/2007/EN have been described previously [26]. BuPV was obtained from P. Kirkland (Elizabeth Macarthur Agriculture Institute, Menangle New South Wales, Australia) and the BDV-1 strain Frijters from A. J. de Smit (Institute for Animal Science and Health, Lelystad, The Netherlands). The BDV-3 strain Gifhorn [29,30], the APPV isolate L277 [31] as well as the pestivirus strain Aydin have been described previously [20]. 

### 2.2. Antisera

TSV isolate 70282/2007/EN, BuPV and BDV strain Frijters-specific antisera were collected during the animal experiment of the present study. For cross NT, one serum sample of the final sampling time point of each infection group was used [animal #3094 (BDV strain Frijters), animal #3085 (TSV isolate 70282/2007/EN) and animal #3086 (BuPV)]. In addition, six porcine antisera were included, that were taken from pigs experimentally infected with the CSFV strains Alfort/187 [genotype (gt) 1.1], Paderborn (gt 2.1) and Diepholz (gt 2.3), BDV strain Gifhorn (BDV-3), pestivirus strain Aydin or APPV. These sera were obtained from the serum sample collection of the EU and OIE Reference Laboratory for CSF. The corresponding animal experiments were made known to the Specialized Department of Animal Welfare Service of the Lower Saxony State Office for Consumer Protection and Food Safety (LAVES; Permit Number: LAVES AZ 08A 538) according to the German animal welfare act.

### 2.3. Preparation of RNA and Real-Time (RT)-PCR

RNA extraction of serum samples was performed using the QIAmp Viral RNA Mini Kit (Qiagen, Hilden, Germany) according to the manufacturers protocol. Viral RNA of TSV and BDV was detected by a pestivirus-specific real-time RT-PCR [32]. For detection of BuPV RNA a previously described RT-PCR was used [33]. 

### 2.4. Complete Genome Sequencing and Phylogenetic Analysis

The genome sequences of TSV isolates 92019/2007/AG and 70282/2007/EN as well as for BDV strain Frijters were determined by next-generation sequencing on an Illumina HiSeq as recently described [34,35]. Sanger sequencing of PCR amplicons was performed to confirm single genomic regions. Pairwise genetic distances (p-distances) were calculated as described previously [1]. Phylogenetic analysis was performed using the Maximum Likelihood method and JTT matrix-based model as described previously and implemented in Mega X [36,37]. For this purpose, amino acid sequences of complete polyproteins were used. Two TSV sequences (isolates 92019/2007/AG and 70282/2007/EN) and the BDV strain Frijters sequences were generated within this study and submitted to NCBI GenBank. Representative sequences of other pestiviruses (*n* = 29) were obtained from GenBank: APPV (GenBank: AWL21794, QDC19463, ASB30697, AUL76968, AUL76967), Aydin (GenBank YP006860588), Bat pestivirus (GenBank AYV99177), BDV (GenBank: AAF02524, AHC08742, ADK63187, AHN82189), BuPV (GenBank YP008992092), BVDV-1 (GenBank AAA42860), BVDV-2 (GenBank ACQ83621), CSFV (GenBank: AFW90184, AAL68894, CAA61161, AAV98354, ALP83489), Giraffe-like pestivirus (GenBank AHW57610), HoBi-like pestivirus (GenBank BAO04453), Linda virus (GenBank YP009407716), ovIT PeV (GenBank QDJ94981), Pangolin pestivirus (GenBank QIE06436), Phocoena pestivirus (GenBank QFQ60724), Pronghorn antelope virus (GenBank YP009026415), Rat pestivirus (GenBank YP009109567), Rodent pestivirus (GenBank ATP66855), Rodent pestivirus (GenBank ATP66856).

### 2.5. Determination of Virus Growth on Different Cell Lines

To investigate the growth efficiency in porcine cells, PK-15, SK6 and STE cells were infected with TSV isolate 70282/2007/EN or CSFV strain Diepholz. In addition, a bovine (MDBK) and an ovine (SFT-R) cell line were included. Briefly, each cell line was seeded in six-well plates one day before infection to reach 80–90% confluency on the day of infection. Cells were infected for one hour with TSV or CSFV using a multiplicity of infection (MOI) of 0.1. Afterwards, the cells were washed six times with phosphate-buffered saline (PBS) and incubated for 72 h at 37 °C. Cell culture supernatants were collected at zero and 72 h post infection (hpi). Infectious titers were determined by endpoint dilution assay in quadruplicates of three independent infection experiments. At 72 h the cells were heat fixated for four hours at 80 °C. Antigen detection was performed by immune-peroxidase staining as described in the Manual of Diagnostic Tests for Detection of CSF, which was composed by the EU and OIE Reference Laboratory for CSF and is available on the website of the EU and OIE Reference Laboratory for CSF (https://www.tiho-hannover.de/kliniken-institute/institute/institut-fuer-virologie/eu-and-oie-reference-laboratory, accessed on 16 June 2021) [38].

To compare in vitro replication of TSV and other pestiviruses, porcine PK-15 and ovine SFT-R cell lines were infected with TSV isolate 70282/2007/EN, CSFV strain Diepholz, BuPV, and BDV strain Frijters as described above. BDV strain Frijters and BuPV are pestiviruses that were isolated from pigs. Cell culture supernatants were collected at 24, 48 and 72 hpi. For TSV, CSFV and BDV detection of viral antigen was performed according to the Manual of Diagnostic Tests for Detection of CSF [38]. The BuPV antigen was detected by immunofluorescence staining using a porcine BuPV-specific antiserum (dilution 1:12,000) in combination with the secondary anti-body Alexa fluor^®^ 594 goat anti-swine IgG (Dianova, 1:1000 dilution). All antibody dilutions were prepared using PBS containing 0.1% Tween_20_. The BuPV-specific antiserum was kindly provided by P. Kirkland (Elizabeth Macarthur Agriculture Institute, Menangle, New South Wales, Australia). 

### 2.6. Animal Experiment

The animal experiment was carried out under high containment conditions in the animal facility of the Research Center for Emerging Infections and Zoonosis, TiHo, Hannover, Germany. Ten piglets (aged 8–10 weeks) were purchased from a commercial breeding farm and randomly allocated into two groups with three animals [inoculation group BDV strain Frijters (animal numbers: 3087, 3088, 3094) and inoculation group BuPV (animal numbers: 3080, 3086, 3093)] and in one group with four animals (inoculation group TSV isolate 70282/2007/EN; animal numbers: 3085, 3090, 3092 and 3096). Animals were kept litterless, fed commercial pig feed and had access to water *ad libitum*. 

Before infection, the animals tested negative for pestivirus genome. After one week of acclimatization, the pigs were inoculated intramuscularly with BDV strain Frijters (10^8.05^ TCID_50_), BuPV (10^8.55^ TCID_50_) or TSV isolate 70282/2007/EN (10^5.55^ TCID_50_), respectively. Virus inocula were confirmed by nucleotide sequencing. The general health of the animals was recorded daily by measuring rectal body temperature and monitoring any clinical signs. Blood samples (full blood and anti-coagulated EDTA blood) were taken at days 0, 3, 5, 7, 14, 28, 42 post infection and before euthanasia. Leucocyte and thrombocyte counts were determined using a hematology analyzer (Abacus Junior vet/130464, Guder Medizintechnik, Germany) at each sampling day. Serum samples were analyzed for pestivirus genome by RT-PCR and for antibodies by NT. At day 28 post infection, all animals were boosted with the respective virus. The pigs were euthanized between 52 or 59 days post infection (dpi).

The corresponding animal experiment was made known to Specialized Department of Animal Welfare Service of the Lower Saxony State Office for Consumer Protection and Food Safety (LAVES; Permit Number: 42502-04-18/2761) according to the German animal welfare act.

### 2.7. ELISA

To determine the interference with the serological CSFV diagnostic, a commercially available CSFV-specific antibody ELISA (CSFV Ab ELISA; IDEXX) was used for the analysis of the experimentally derived serum samples. The protocol follows the manufacturers´ instructions. 

### 2.8. Cross-Neutralization Test

Virus neutralization was performed as described in the protocol of the Manual of Diagnostic Tests for Detection of CSF [38] using different CSFV strains (Alfort/187, Paderborn and Diepholz), two BDV strains isolated from pigs (BDV-1 strain Frijters and BDV-3 strain Gifhorn), the TSV isolate 70282/2007/EN, pestivirus strain Aydin, the APPV isolate L277, and BuPV. The titration of the corresponding antisera with each virus started with a 1:5 dilution up to a 1:10,240 dilution. 

Antigen detection was performed by immune-peroxidase staining as described above (paragraph 2.5 Determination of virus growth on different cell lines). A sample is classified positive, if the antibody titer is ≥10 ND_50_.

The antigenic relatedness was determined by the calculation of the coefficient of antigenic similarity (*R*) using the following formula [39]:

(1) $$\;R=100\times \sqrt{\frac{titer\;strain\;A\;with\;antiserum\;B\times titer\;strain\;B\;with\;antiserum\;A}{titer\;strain\;A\;with\;antiserum\;A\times titer\;strain\;B\;with\;antiserum\;A\;}}$$

The NT is a sensitive and reliable test to detect antibodies against CSF and is applied for differential diagnosis with regard to cross-reacting antibodies generated after infections of pigs with ruminant pestiviruses. So far, to differentiate between infections of pigs with CSFV or with ruminant pestiviruses a three-fold difference or more between end-points of two titrations are recommended by the World Organization of Animal Health (OIE; Manual of Diagnostic Tests and Vaccines for Terrestrial Animals 2021; chapter 3.9.3.) [40]. Since variations of two to three titer steps can be observed by repetition of VNTs, differences of three titer steps or below do not allow a clear differentiation between infections with CSFV and ruminant pestiviruses [41]. 

## 3. Results

### 3.1. Genetic Characterization

So far, short fragments of the N^pro^ encoding region (GenBank U80905) and the 3′ non-translated region (NTR) (GenBank AF037410) were used to determine the genetic relatedness of BDV-1 strain Frijters to other pestiviruses [13,32]. In the current study, the complete genome sequence of BDV-1 strain Frijters was determined and used for genetic characterization (GenBank MZ664275). The genome has a length of 12,329 nucleotides (nt) comprising an open reading frame (ORF) of 11,688 nt which encodes for a polyprotein of 3895 amino acids. The 5′ NTR is 372 nt and the 3′ NTR is 269 nt long. The complete genome sequence of BDV-1 strain Frijters shares 92% identity with the genome of BDV-1 strain FNK2012-1, which was the first BDV isolated from a healthy fattening pig in Japan in 2012 (GenBank AB897785) and 90% with the genome sequence of BDV-1 reference strain X818 [9,42]. 

Moreover, for genetic characterization of TSV the complete genomes of both TSV isolates, 92019/2007/AG and 70282/2007/EN [26] were determined by high throughput sequencing (HTS). For the genome sequence of isolate 92019/2007/AG, the 5′ extended sequence obtained by HTS could not be verified by PCR. Thus, the genome start was determined by multiple sequence alignment using sequences of closely related pestiviruses and trimmed accordingly. The genome of TSV isolate 92019/2007/AG has a length of 12,286 nt (GenBank MZ664274). For isolate 70282/2007/EN, the genome ends were not successfully sequenced and a few nucleotides were lacking (24 nt at 5′ end, 26 nt at 3′ end; GenBank MZ664273). Both sequences share a high identity showing a pairwise distance (p-distance) of only 0.08 in the ORF comprising 11,685-nt which encodes for a polyprotein of 3894 amino acids. Showing nucleotide p-distances of 0.23 in the ORF, genomes of TSV share highest similarity to genomes of ovine pestiviruses (GenBank MK618726, MG770617, MK618725) discovered in Italy in 2017 [4]. Similar p-distances of 0.24 were calculated by comparison to most closely related CSFV strains (e.g., CSFV Eystrup, GenBank AF326963). In contrast, nucleotide p-distances to BDV strains Gifhorn (GenbanK GQ902940) and Frijters (this study) were 0.28 and thus significantly higher. The 5′ NTR of TSV has a length of 374 nt and the 3′ NTR comprises 227 nt. The previously determined partial 5′ NTR sequence of isolate 92019/2007/AG (GenBank KU856552) is identical to the sequence determined in the present study, whereas the 5′ NTR sequence of isolate 70282/2007/EN (GenBank KU856551) showed one mismatch compared to the newly determined sequence [26]. Interestingly, for the 5′ NTR sequences the absolute identity was up to 87.5% (94% coverage) to the new ovIT PeV (GenBank MG770617) and up to 85.6% (100% coverage) to diverse CSFV strains (e.g., GenBank LT593748). Similar absolute identities were observed for the 3′ NTR when comparing TSV and CSFV (up to 86% identity with 85% coverage). No closer related 3′ NTR sequence could be found. One reason for this might be the incomplete genome sequences available for the new ovIT PeV (GenBank MG770617, MK618725, MK618726).

**Figure 1:**
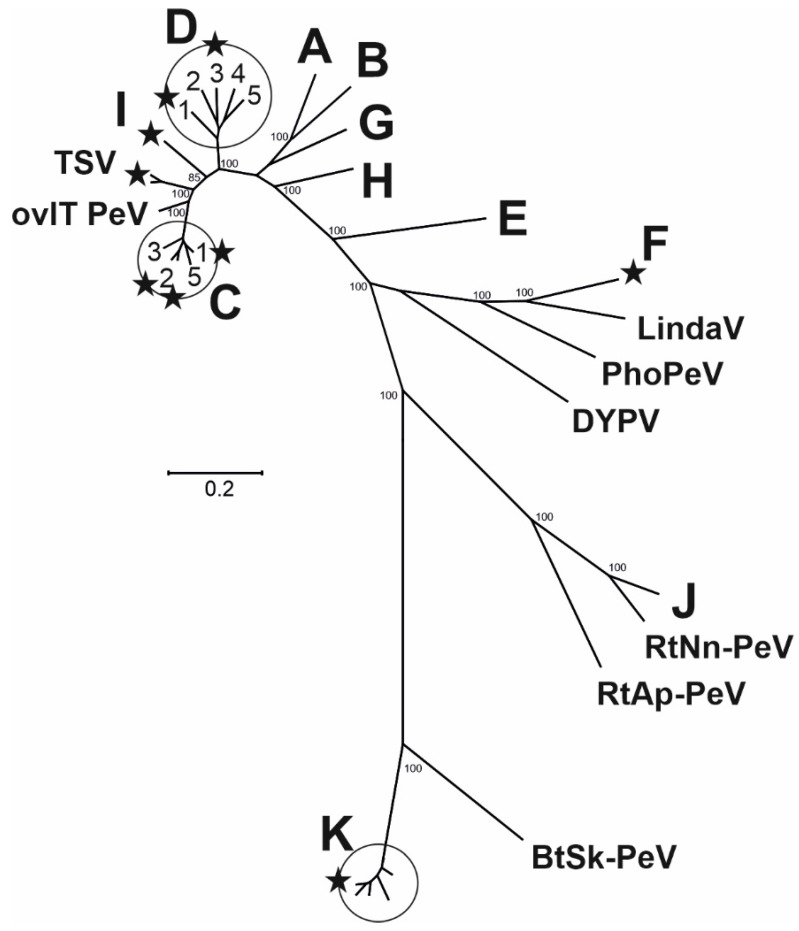
Genetic relatedness of pestiviruses. Phylogenetic analysis based on 32 polyprotein sequences of approved pestivirus species and recently described unassigned pestiviruses having a length ranging between 3604 amino acids (*Pestivirus K*, GenBank AUL76968) and 4026 amino acids (*Pestivirus J*, GenBank YP009109567). Analysis was performed by Maximum Likelihood method using the Mega X software. For statistical analysis, the tree was calculated with 100 repetitions. Bootstrap values of ≥70 are indicated only for the main nodes that define pestivirus species. Established species (*Pestivirus A–K*) are indicated by letter code as proposed previously [1]. Unassigned pestivirus sequences are indicated by abbreviations of virus names (ovine Italy pestivirus, ovIT PeV; Tunisian sheep –like pestivirus, TSV; Linda virus, LindaV; Phocoena pestivirus, PhoPeV; Dongyang pangolin virus, DYPV; rodent *Niviventer niviventer* pestivirus, RtNn–PeV; rodent *Apodemus peninsulae* pestivirus, RtAp–PeV; bat *Scotophilus kuhlii* pestivirus, BtSk–PeV). Numbers in the circles refer to defined CSFV and BDV genotypes of the species *Pestivirus C* and *Pestivirus D*, respectively. Isolate names and GenBank accession numbers of used sequences are listed in the materials and methods section. Asterisks indicate the isolates used for cross-neutralization tests in this study.

For further characterization, a phylogenetic analysis of the complete polyprotein sequences of the two TSV isolates 92019/2007/AG and 70282/2007/EN, BDV strain Frijters as well as members of the established pestivirus species and newly detected pestiviruses was performed. The two TSV isolates, pestivirus strain Aydin, and the novel ovIT PeVs formed three distinct groups located between CSFV and BDV. The phylogenetic group of TSV is located between the novel ovIT PeV and pestivirus strain Aydin (Figure 1). A previous study showed that infections of pigs with Aydin virus did not result in disease [20]. Since the genetic relationship of TSV and CSFV is even closer than between Aydin pestivirus and CSFV, viral replication of TSV was further characterized on porcine cells and after experimental infection of pigs.

### 3.2. Virus Growth on Different Cell Lines

Due to the close genetic relatedness of TSV to CSFV, it was of particular interest to investigate whether TSV could replicate in porcine cells. Against this background, three porcine epithelial cell lines (PK-15, STE and SK6) were tested for their permissivity to TSV in comparison to CSFV. In addition, the ovine SFT–R and bovine MDBK cell lines were used. 

**Figure 2:**
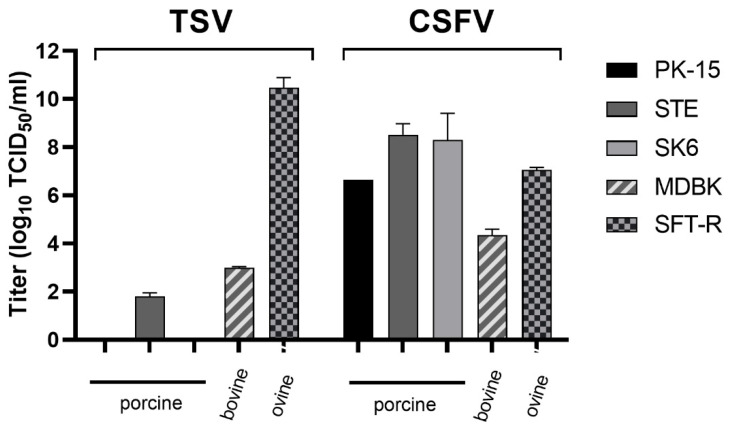
Viral replication of Tunisian sheep-like virus (TSV isolate 70282/2007/EN) and classical swine fever virus (CSFV strain Diepholz) on different cell lines using a multiplicity of infection (MOI) of 0.1. The virus titers were determined as 50% tissue culture infectious doses (TCID_50_) per mL at 72 hours post infection. The porcine kidney cell lines PK-15 and SK6 as well as the swine testis cell line STE were used. Additionally, the bovine MDBK and the ovine SFT-R cell line were included to characterize the permissivity.

TSV replicated very efficiently and reached a remarkable high titer in SFT–R cells (10^10.47^ TCID_50_/mL), which demonstrated that this virus is well adapted to ovine cells (Figure 2). In comparison to this, the propagation of TSV on porcine STE cells (10^1.8^ TCID_50_/mL) and on bovine MDBK cells (10^2.9^ TCID_50_/mL) was very inefficient. Moreover, no virus replication was detected in the porcine cell lines PK-15 and SK6 (Figure 2). CSFV replicates on porcine as well as on non-porcine cell lines to various extents [10^4.34^ TCID_50_/mL (MDBK cells) up to 10^8.52^ TCID_50_/mL (STE cells)]. Among the porcine cell lines, replication of CSFV was less efficient on PK-15 cells compared to SK6 and STE cells. Interestingly, CSFV reached similar titers on PK-15 and ovine SFT-R cells (Figure 2). 

**Figure 3:**
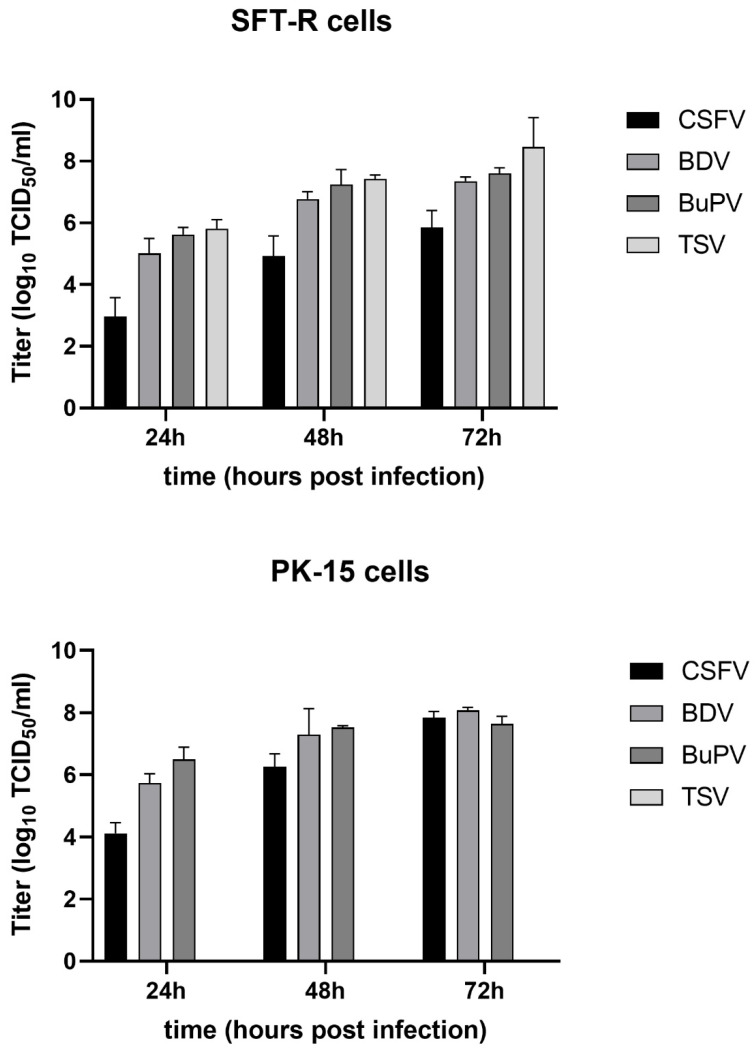
Kinetic of viral replication of Tunisian sheep-like virus (TSV isolate 70282/2007/EN), classical swine fever virus (CSFV strain Diepholz), border disease virus (BDV strain Frijters) and Bungowannah virus (BuPV) on ovine (SFT-R) and porcine (PK-15) cells. The cells were infected using a multiplicity of infection (MOI) of 0.1. Infectious titers were determined as 50% tissue culture infectious doses (TCID_50_) per mL at the indicated time points post infection by endpoint dilution assay in quadruplicates from cell culture supernatants of three independent infection experiments. Mean values with standard deviations are shown.

In addition, replication of TSV and CSFV was analyzed in comparison to BDV strain Frijters, which was isolated from pigs, and BuPV, respectively. For this purpose, viral infections were performed on the porcine cell line PK-15 and the ovine cell line SFT-R. Viral titers were determined 24, 48 and 72 hpi (Figure 3). At 24 hpi, comparable infectious titers were observed on SFT-R cells for TSV (10^5.8^ TCID_50_/mL), BDV (10^5.0^ TCID_50_/mL), and BuPV (10^5.6^ TCID_50_/mL). However, after 72 hpi, the highest viral titer (10^8.46^ TCID_50_/mL) was detected for TSV. BDV and BuPV showed infectious titers of approximately 10^7^ TCID_50_/mL. In comparison to this, again no infectious TSV was detected on PK-15 cells, whereas BDV and BuPV replicated efficiently on PK-15 cells by reaching infectious titers up to 10^7^ TCID_50_/mL at 72 hpi. These titers are comparable to those obtained for CSFV (Figure 3). 

### 3.3. Experimental Infection of Pigs

So far, infection of pigs with TSV and a possible impact on pig health have not been studied. To evaluate the in vivo replication and to assess the pathogenicity of TSV in comparison to BDV and BuPV an infection study with ten piglets was performed. 

The present study revealed that pigs could be infected by all three pestiviruses, including TSV. After infection, none of the animals developed fever or showed any clinical signs. In addition, counts of leucocytes and thrombocytes in all animals remained within physiological range (data not shown). To monitor viral replication of the three pestiviruses, blood samples were collected at 3, 5, 7, and 14 dpi and were analyzed by RT-PCR. Across all study groups, only low viral replication was observed. Although TSV replication on porcine cells was highly inefficient, the TSV genome was detected in the blood of inoculated pigs at 5, 7 and 14 dpi in at least one out of four piglets (Cq value: 35.9–39.0) confirming that under experimental conditions TSV can replicate in domestic pigs. Moreover, viral replication was comparable to the replication of the other two pestiviruses (Table 1). The piglets, which were infected with BuPV, tested positive from 3 to 14 dpi (Cq values: 33.8–39) (Table 1). Similar results had been reported [43]. Genomes of BDV strain Frijters were detected at 5 dpi (Cq value: 36.1) for one out of three animals. 

In addition, all inoculated animals tested positive for neutralizing antibodies in NT using the homologous virus, which confirms that all animals seroconverted (Table 2). 

### 3.4. Antigenic Relatedness

The analysis of antigenic relatedness was performed by cross NT. In total, nine pestivirus strains and the corresponding porcine sera obtained after infection with these virus strains were analyzed. Three CSFV strain were used comprising one genotype 1 (Alfort/187) and two genotype 2 isolates (Paderborn, Diepholz). Genotype 2 isolates caused the most recent outbreaks in the European Union [44]. In addition, the BDV strains Frijters and Gifhorn [14,30], BuPV, APPV strain L277, as well as TSV isolate 70282/2007/EN and Aydin virus, which are genetically closely related to CSFV, were included in the cross NT. 

In general, the highest titers of neutralizing antibodies were detected against the homologous viruses (Table S1). To determine the antigenic relatedness between the pestiviruses studied here, the antibody titers were used for the calculation of the coefficient of antigenic similarity (*R*) (Table S2). *R* values ≤ 25 represented significant differences (≥ 4-fold) in titers of homologous and heterologous antisera [29]. 

**Figure 4:**
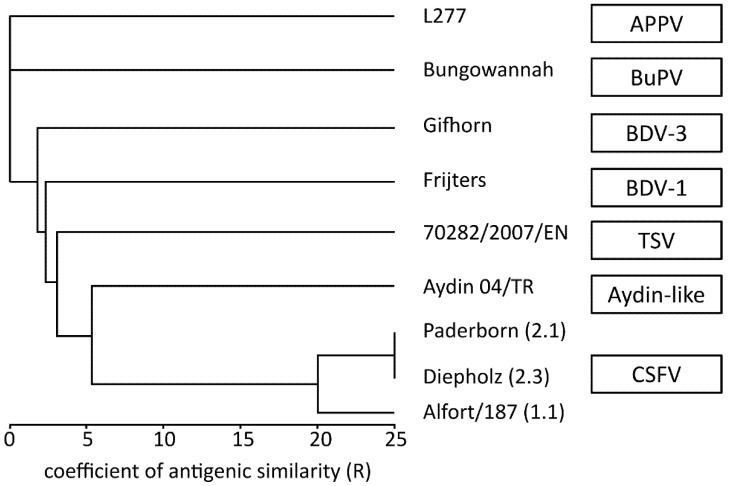
Antigenic relatedness of pestiviruses, including Tunisian sheep-like virus (TSV, isolate 7082/2007/EN), the two border disease virus (BDV) strains Frijters and Gifhorn, classical swine fever virus (CSFV) strains Alfort/187, Paderborn and Diepholz, Aydin virus (isolate Aydin 04/TR) as well as Bungowannah pestivirus (BuPV), and atypical porcine pestivirus (APPV) isolate 277. The antigenic tree based on coefficients of antigenic similarity (*R*). *R* values ≤ 25 represent significant differences (≥4-fold) in titers of homologous and heterologous antisera. *R* values of >25 are not drawn to scale.

The dendrogram of antigenic relatedness confirmed that the tested CSFV strains were grouped together (Figure 4). However, the present analysis indicated antigenic differences between CSFV strain Alfort/187 and Paderborn, whereas both viruses belong to one pestivirus species (Table S1). It has already been mentioned that differences in the E2 protein sequence can influence cross-neutralization with sera from heterologous CSFV strains [45]. The dendrogram showed that the pestivirus strain Aydin and TSV are antigenically more closely related to CSFV (averaged *R* value for the Aydin pestivirus and the three CSFV strains = 5.4; averaged *R* value for TSV and the three CSFV strains = 3.9) than to BDV (averaged *R* value for Aydin pestivirus and the two BDV strains = 2.1; averaged *R* value for TSV and the two BDV strains = 2.1), respectively (Table S2). For BuPV and APPV, no cross-reactivity with any of the other tested pestivirus strains was observed (Table S2; Figure 4).

### 3.5. Interference with Serological CSF Diagnosis

Because of the close genetic and antigenic relatedness of TSV to CSFV, it was interesting to investigate a possible interference of the serological CSF diagnosis by cross-reactive antibodies induced after infection with TSV. Both, ELISAs and NTs were used for serological diagnosis of a CSFV infection. The performance of NTs was of particular importance because they are used as confirmatory test in case of positive or doubtful ELISA results and for differential diagnosis. 

To assess the cross-reactivity in CSFV-specific assays, serum samples of the individual animals infected with TSV isolate 70282/2007/EN, BDV strain Frijters, and BuPV were analyzed by NT using CSFV (Alfort/187 and Diepholz), BDV (Moredun), and BVDV (NADL) reference strains that are applied frequently for differential diagnosis. In addition, the homologous viruses were used as test virus in the NT (Table 2). 

**Figure 5:**
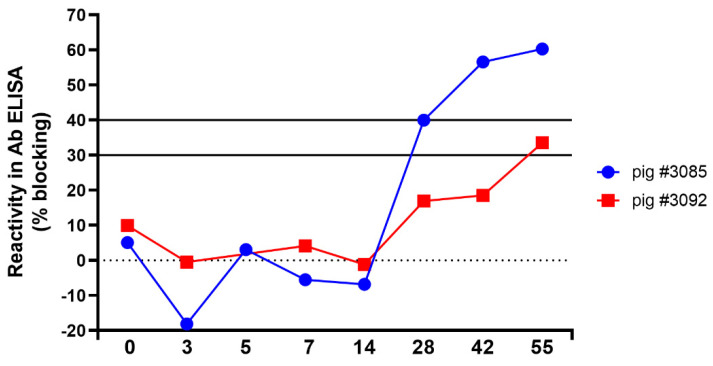
Detection of cross-reactive antibodies. Analysis of serum samples from two animals (#3085 and #3092) collected at the indicated time points after infection with Tunisian sheep-like virus isolate 70282/2007/EN using a commercial CSFV E2-specific antibody ELISA.

Samples that were taken from pigs infected with TSV tested positive in the NT using the CSFV strain Diepholz. Two of these samples showed a titer of 160 ND_50_ (animal no.: #3085 and #3092) and also tested positive with lower antibody titers in the Moredun- (ND_50_ = 20 or 10) and NADL-specific NT (ND_50_ = 20). Even if the homologous virus was used in the NT, only a three- or four-fold difference in the antibody titers against CSFV strain Diepholz (ND_50_ = 160) and TSV (ND_50_ = 480 or 640) was detected (Table 2). Additionally, these sera tested positive (#3085) and doubtful (#3092) in the CSFV E2-specific antibody ELISA. For both animals, a clear increase of cross-reactive antibodies was detected in the CSFV-specific ELISA at 14 dpi (Figure 5). 

Serum samples of the remaining two animals of the TSV infection group (animals: #3090 and #3096) tested positive in the NT using the CSFV strain Diepholz, but negative in the NT using CSFV strain Alfort/187 and in the CSFV-specific ELISA. Taken together, no clear differentiation between CSFV and TSV was possible for both samples with a doubtful or positive ELISA result. 

Samples derived from animals infected with BDV strain Frijters showed also cross-reactivity with CSFV strain Diepholz. Remarkably, the titers of neutralizing antibodies against BDV strain Moredun were 8- to 16-fold lower than the neutralizing activity against CSFV Diepholz. The highest neutralizing antibody titers were detected against the homologous virus, BDV strain Frijters. In comparison to these titers, the antibody titers against CSFV strain Diepholz are 4- (difference of two titer steps) to 5.3-fold lower (difference of 2.3 titer steps) (Table 2). Using the difference of three titer steps to interpret the results, a clear differentiation of CSFV and BDV was not possible even when the homologous virus was included in the NT. Low cross-reactivity was also observed with BVDV-1 strain NADL and CSFV strain Alfort/187 (Table 2). In addition, all samples tested negative in the CSFV-specific ELISA (Table 2). 

All sera taken from animals infected with BuPV tested negative in the CSFV-specific ELISA as well as in the NTs using CSFV (Table 2). 

## 4. Discussion

Pigs can serve as hosts for different porcine pestiviruses, including the widely distributed CSFV and APPV [17,18], the unique pestiviruses BuPV and Linda virus [6,19], as well as various ruminant pestiviruses [7,8,9,10,11,12,13,14,15,16]. Natural infections of pigs with BVDV-1, BVDV-2, and BDV occasionally occur [7,8,9,10,11,12,13,14,15,16], and one illustrative example is represented by the detection of the BDV strain Frijters from pigs during the course of CSF sero-surveillance studies [14]. Interestingly, a close genetic relatedness to CSFV was described for three additional groups of ruminant pestiviruses detected in sheep or goats, namely Aydin-like pestiviruses [20], the recently identified group of ovine and caprine pestiviruses from Italy [4,21,22] and TSV [25,26]. So far, complete genome sequences of TSV are not available and therefore phylogenetic studies on TSV were limited to analysis of partial genomic and deduced amino acid sequences (N^pro^-E2 sequence) [25]. In the present study, complete polyprotein encoding sequences of two TSV isolates (92019/2007/AG and 70282/2007/EN [26]) were determined and used for phylogenetic analysis. The results obtained by this analysis confirmed the previously reported close genetic relatedness of TSV to CSFV. In addition, to expand the available data on pestiviruses isolated from pigs, the complete genome sequence of BDV strain Frijters was established. The phylogenetic analysis based on complete polyprotein sequences showed that the two TSV isolates form a distinct phylogenetic group that is more closely related to CSFV than to BDV or other ruminant pestiviruses (Figure 1). Furthermore, TSV is located between the pestivirus strain Aydin and the recently described group of ovIT PeV. The close relationship to the latter group of pestiviruses from Italy as well as to CSFV fits well to the hypothesis that CSFV emerged from TSV by a host switch from sheep to pigs and that CSFV and ovIT PeV share a common ancestor closely related to TSV [22]. 

Even though these three groups of small ruminant pestiviruses represent the genetically closest relatives of CSFV, which solely infects pigs, field infections of pigs with TSV, Aydin-like pestiviruses and ovIT PeV had not been described so far. However, it was shown that pigs can be experimentally infected with the Aydin pestivirus without causing clinical disease and with the ovIT PeV associated with only mild clinical signs in pigs. For all experimentally infected pigs, a strong seroconversion was detected [20,23]. Considering that the genetic relatedness of TSV to CSFV is even closer than between Aydin pestivirus and CSFV, it was of particular interest to analyze TSV infection *in vitro* on different porcine cell lines as well as *in vivo* in domestic pigs and to assess its potential role as a pathogen for pigs. 

The results of the present study showed that TSV replicates highly efficiently on ovine (SFT-R) cells and produced very high infectious virus titers. Despite the close genetic relationship to CSFV, TSV was not able to replicate on two porcine cell lines (PK-15 and SK6) and produced only low virus titers on porcine STE cells (Figure 2 and Figure 3). In contrast, BDV strain Frijters, BuPV and CSFV strain Diepholz, showed only low or no significant titer differences on ovine SFT-R cells and the three porcine cell lines. It is remarkable that TSV replicates up to a titer of 10^10,47^ TCID_50_/mL on ovine cells. Such an efficient replication in cell culture has not been described for other pestiviruses and highlights that TSV infection and replication is well adapted to the ovine, but not to the porcine host. However, despite of the lack of efficient virus replication on porcine cells, pigs were successfully infected with TSV by the intramuscular route. Low viral genome loads were sporadically detected at 5, 7 and 14 dpi in at least one out of the four TSV infected pigs. A similar low viral replication efficiency was also observed for pigs infected with BDV strain Frijters and BuPV. Both viruses were not able to induce apparent clinical symptoms after horizontal infection of pigs, but a disease could develop after in utero infection of the fetus [14,43]. The current experiment demonstrated that infections of weaner-aged pigs with TSV are comparable to infections with BuPV and BDV strain Frijters with regard to low viral genome loads in the blood and the absence of clinical disease. In the natural host (e.g., lambs), TSV can cause mild fever and leucopoenia three to six days post infection. After transplacental infection of pregnant ewes with TSV, clinical signs typically described for border disease were reported, including a high rate of abortion, still birth as well as the birth of weak and hairy-shaker lambs [24]. Unlike infections with CSFV, horizontal infections of pigs with ruminant or other porcine pestiviruses are often subclinical and severe consequences usually occur only when pregnant sows are infected [11,43,46,47]. The outcome of intrauterine infections is dependent on the stage of gestation at which the infection occurs. Infections with the ruminant pestiviruses BVDV and BDV can be pathogenic for porcine fetuses, whereby the pathogenicity seems to be strain dependent [11]. An infection of the fetus can cause malformations, fetal death or congenital disorders as well as persistent infections in the offspring. Such persistently infected animals have a major impact on the transmission and spread of the disease since these animals are viremic, antibody negative, and constantly excrete large amounts of viruses [8,11,46]. Even if TSV is apparently not able to induce disease in pigs after horizontal infection, the consequences of vertical infection for the porcine fetus remain unknown and need to be analyzed in future studies. In this context, it will also be interesting to learn whether intrauterine infections with TSV can cause persistent infections in piglets. 

A major aim of the present study was to characterize the antigenic relationship of TSV to selected other pestiviruses by a cross NT. For this purpose, TSV, three CSFV strains, two BDV strains isolated from pigs, Aydin pestivirus, BuPV, and APPV were analyzed together with the corresponding porcine sera produced after infection of pigs with these viruses. The current study revealed a remarkable cross-reactivity of TSV specific sera with CSFV and showed that TSV is antigenically more closely related to CSFV than to BDV (Table 2, Figure 4). Thus, the previously reported conclusion that TSV is antigenically more similar to BDV than to CSFV [25] is not supported. A reason for this discrepancy might be that cross NT in the previous study was performed by using only one CSFV strain (Alfort/187; genotype 1.1), which is an old laboratory reference strain. Moreover, the TSV-specific sera in that study were collected from the ovine host [25]. 

Even though a broad range of various pestiviruses was tested in a cross NT, no neutralizing antibody titers of BuPV-specific sera were detected for any of the other pestiviruses or *vice versa*. This confirmed previous reports on the lack of cross-neutralization against the pronghorn pestivirus as well as the BVDV and BDV-1 strains, which circulate in Australia [19]. Since the establishment of a cell culture system for infection studies with APPV was challenging, the antigenic relatedness of APPV to other pestiviruses was addressed for the first time in the current study. For the investigated set of various pestiviruses, apart from the homologous virus, no neutralizing activity of the APPV-specific antisera was observed or *vice-versa*: APPV was not neutralized by sera produced after infection with these heterologous, only distantly related, pestiviruses. This is in line with a previous study in which no interference of APPV-specific antibodies with CSFV-specific serological diagnostic tests was detected [31]. 

As a consequence of the close antigenic relatedness of TSV to CSFV, cross-reactivity in CSFV-specific antibody assays (ELISA and NT) was observed. One serum tested positive and one tested doubtful in the CSFV-specific ELISA. Even if the homologous virus had been included in the NT, for both sera the differences in the antibody titers against TSV and CSFV were only 3- to 4-fold (corresponding to a difference of 1.5 to two titer steps) (Table 2). Such low differences are not sufficient for a clear discrimination between CSFV and TSV. Comparable results were previously reported for Aydin pestivirus [20] and the ovIT PeV [21,22,31], which are also genetically and antigenically more closely related to CSFV than to BDV. Moreover, a high level of cross-reactivity against CSFV reference strain Diepholz was also detected for sera from BDV strain Frijters infected pigs. Again, on the basis of the results detected by NT, a differentiation between CSFV and BDV was not possible, even if the homologous virus was included as test virus for NT. However, analysis of sera from pigs infected with BDV strain Frijters by a commercial CSFV E2 antibody ELISA tested negative. Furthermore, the present study demonstrates that the titers against the BDV strain Moredun, which is frequently used as test virus for differential diagnosis, were significantly lower in comparison to the titers detected against the CSFV strain Diepholz. This result emphasizes that, with regard to differential diagnosis, using more than one BDV or BVDV strain as a test virus in NT is recommended, as well as including ruminant pestivirus isolates that are representative for the country or region.

## 5. Conclusions

The TSV isolates from small ruminants form a distinct phylogenetic group more closely related to *Pestivirus C* (CSFV) than to *Pestivirus D* (BDV). Pigs are susceptible to TSV, but did not show any clinical signs after horizontal infection. However, consequences after *in utero* infection of the porcine fetus remain unknown and need to be addressed in future studies. Due to the close antigenic similarity to CSFV, field infections of pigs with TSV and other ruminant pestiviruses can significantly interfere with serological CSF diagnosis. Moreover, the transmission of TSV from small ruminants to porcine hosts might result in serious consequences for CSF control and surveillance.

## Figures and Tables

**Table 1:** Detection of viral RNA by real-time RT-PCR in serum samples of piglets at different days after infection with the indicated viruses.

Virus	Animal Number	Cq Values				
		0 dpi ^1^	3 dpi	5 dpi	7 dpi	14 dpi
TSV	3085	-	-	38.9	35.9	39.0
TSV	3090	-	-	-	38.6	n.t. ^2^
TSV	3092	-	-	n.t.	36.1	-
TSV	3096	-	-	-	-	n.t.
BuPV	3080	-	n.t.	n.t.	35.6	-
BuPV	3086	-	35.6	33.8	36.3	-
BuPV	3093	-	38.3	34.6	36.0	39.0
BDV	3087	-	-	36.1	-	-
BDV	3088	-	-	-	-	-
BDV	3094	-	-	-	n.t.	-

^1^ dpi = days post infection; ^2^ n.t. = not tested. No sample material was available.

**Table 2:** Cross-reactivity of sera taken from the individual animals at the end of the experiment. The samples were analyzed by a CSFV E2 antibody ELISA and by NTs against the indicated viruses.

Virus	Animal Number	CSFV ELISA	NT ^4^				
			BVDV NADL	BDV Moredun	CSFV Alfort/187	CSFV Diepholz	Homologous Virus
TSV	3085	pos. ^1^	20	20	-	160	640
TSV	3090	neg. ^2^	-	-	-	40	240
TSV	3092	dbtf. ^3^	15	10	-	160	480
TSV	3096	neg.	-	-	-	30	640
BDV	3087	neg.	10	30	-	240	1280
BDV	3088	neg.	-	20	10	320	1280
BDV	3094	neg.	40	30	20	480	1920
BuPV	3080	neg.	-	-	-	-	2560
BuPV	3086	neg.	-	-	-	-	6400
BuPV	3093	neg.	-	-	-	-	3200

^1^ = positive; ^2^ = negative; ^3^ = doubtful; ^4^ The titer of neutralizing antibodies is depicted as the reciprocal of the highest serum dilution capable of completely neutralizing 100–300 TCID_50_ of the respective virus strain; - = negative.

## Data Availability

Not applicable.

## Supplementary Materials

The following supplementary tables are available online at https://www.mdpi.com/article/10.3390/v13081539/s1, Table S1: Titer of neutralizing antibodies against different pestivirus strains. Table S2: Antigenic similarity (R).
