# Supplementary material for: Comparative Analysis of Tunisian Sheep-like Virus, Bungowannah Virus and Border Disease Virus Infection in the Porcine Host
Source: Viruses. 2021 Aug 4;13(8):1539. doi: 10.3390/v13081539 (PMC8402848; doi:10.3390/v13081539)
Supplement: Supplementary file 1 [file viruses-13-01539-s001.zip › Table S1.pdf]

viruses MDPI

Table S1. Titer of neutralizing antibodies against different pestivirus strains.

Antiserum against	Pestivirus Isolate								
	**CSFV Alfort/187**	**CSFV Paderborn**	**CSFV Diepholz**	**BDV Frijters**	**BDV Gifhorn**	**TSV 70282/2007/EN**	**Aydin 04/TR**	**Bungowannah**	**APPV L277**
CSFV[1] Alfort/187	**1280** [1]	160	453	17	-	20	24	-	-
CSFV Paderborn	48	**1076**	1280	57	20	14	24	-	-
CSFV Diepholz	320	320	**1076**	17	17	34	160	-	-
BDV[2] Frijters	14	67	320	**3044**	113	24	57	-	-
BDV Gifhorn	17	95	640	37	**7241**	57	95	-	-
TSV[3] 70282/2007/EN	-	67	113	12	67	**640**	14	-	-
Aydin 04/TR	20	17	57	17	17	-	**640**	-	-
Bungowannah	-	-	-	-	-	-	-	**12178**	-
APPV[4] L277	-	-	-	-	-	-	-	-	**67**

[1] The titer of neutralizing antibodies is depicted as the reciprocal of the highest serum dilution capable of completely neutralizing 100 – 300 $TCID_{50}$ of the respective virus strain; Bold = titer of the homologous virus; - = negative; [1] CSFV = classical swine fever virus; [2] BDV = border disease virus; [3] TSV = Tunisian sheep-like virus; [4] APPV = atypical porcine pestivirus.
