# Supplementary material for: Comparative Analysis of Tunisian Sheep-like Virus, Bungowannah Virus and Border Disease Virus Infection in the Porcine Host
Source: Viruses. 2021 Aug 4;13(8):1539. doi: 10.3390/v13081539 (PMC8402848; doi:10.3390/v13081539)
Supplement: Supplementary file 1 [file viruses-13-01539-s001.zip › Table S2.pdf]

viruses MDPI

Table S2: Antigenic similarity (*R*).

**Pestivirus isolate**	**CSFV Paderborn**	**CSFV Diepholz**	**BDV Frijters**	**BDV Gifhorn**	**TSV 70282/2007/EN**	**Aydin 04/TR**	**Bungowan-nah**	**APPV[4] L277**
CSFV[1] Alfort/187	7.5	32.4	0.8	0.1	0.5	2.4	> 0.1	0.3
CSFV Paderborn		59.5	3.4	1.6	3.7	2.4	> 0.1	0.4
CSFV Diepholz			4.1	3.7	7.5	11.5	> 0.1	0.4
BDV[2] Frijters				1.4	1.2	2.2	> 0.1	0.2
BDV Gifhorn					2.9	1.9	> 0.1	0.1
TSV[3] 70282/2007/EN						0.6	> 0.1	0.5
Aydin 04/TR							> 0.1	0.5
Bungowannah								0.1

[1] CSFV = classical swine fever virus; [2] BDV = border disease virus; [3] TSV = Tunisian sheep-like virus; [4] APPV = atypical porcine pestivirus.
